# Tradition to Pathogenesis: A Novel Hypothesis for Elucidating the Pathogenesis of Diseases Based on the Traditional Use of Medicinal Plants

**DOI:** 10.3389/fphar.2021.705077

**Published:** 2021-10-25

**Authors:** Kenny Kuchta, Silke Cameron

**Affiliations:** ^1^ Forschungsstelle für Fernöstliche Medizin, Department of Vegetation Analysis and Phytodiversity, Albrecht von Haller Institute of Plant Sciences, Georg August University, Göttingen, Germany; ^2^ Clinic for Gastroenterology and Gastrointestinal Oncology, University Medicine Göttingen, Göttingen, Germany; ^3^ Clinic, Hann. Münden, Germany

**Keywords:** traditional medicine, pharmacology, pathogenesis, mechanism of action, Evolution, Cultural evolution

## Abstract

Traditional medicines embody knowledge on medicinal plants that has been accumulated through cultural evolution over millennia. In the latter half of the 20th century, two approaches to medicinal plant research have been established: the “Bench to Bedside” and the “Bedside to Bench” approaches which serve primarily for the development of more efficient therapeutics. Here, we propose a third, novel approach: from “Tradition to Pathogenesis” which aims to understand the pathogenesis of diseases based on the cultural evolution of their respective empirical treatments. We analyse multiple examples of diseases where the acting mechanism of traditional treatments across multiple cultures points to the pathogenesis of the respective disease. E.g., many cultures traditionally treat rheumatism with anti-bacterial botanical drugs, which is at odds with our current understanding that rheumatism is an aseptic inflammation. Furthermore, gastric ailments have traditionally been treated with anti-infectious botanical drugs indicating local infections, as demonstrated by the discovery of *Helicobacter pylori* as a common cause of gastric ulcer. Understanding traditional treatments can thus help to elucidate the pathogenesis of the disease.

## Co-Evolution of Traditional Medicine and Medicinal Plants

Recent anthropological research data have led to the astounding conclusion that traditional herbal medicine has most probably a longer history than mankind: Apes have been observed to use medicinal plants for the treatment of diseases (Huffman, 2001). Moreover, human populations that settled in the same region of Africa use the same plants with very similar indications (Huffman, 2001). One example for this transfer of medicinal knowledge from animals to humans is *Vernonia amygdalina* Del. Chimpanzees (*Pan troglodytes*) have been observed on numerous occasions to chew on the bitter pith of this plant as self-medication in case of parasitic nematode infections (Huffman, 2001). Traditional healers of the WaTongwe people of the Mahale Mountains in Tanzania, where the use of *V. amygdalina* by Chimpanzees has also been observed, use this plant for intestinal parasites, diarrhoea, and stomach upset. Phytochemical research has demonstrated that sesquiterpene lactones in *V. amygdalina* possess anthelmintic, antiamoebic, antitumor, and antibiotic properties (Huffman, 2001).

We can thus propose a long-term co-evolution between man and his food and medicinal plants, resulting in the adaption of human pharmacology to the bioactive plant metabolites. The fact that already Neanderthals 50.000 years ago used yarrow (*Achillea millefolium*) and camomile (*Matricaria chamomilla*) - two plants still registered as medicinal plants in the European Pharmacopoeia - as well as poplar buds (*Populus spec.*) (Hardy et al., 2012; Weyrich et al., 2017) as medicine, demonstrates that contemporary phytotherapeutic practice goes back to the dawn of man. The accumulated body of knowledge (referred to as “tradition” or “culture”) is transferrable from person to person as humans and their closest relatives are further able to learn successful behaviours. The process of the improvement and distribution of this knowledge can be referred to as “cultural evolution.” The evolutionary pressure that drives this cultural evolution is the survival benefit for tribes with knowledge of effective treatments. Just as for the use of single herbs, their traditional combinations evolved over time. Various prescriptions include the same medicinal plants in different combinations as the individual effects i.e., anti-inflammatory, mucoprotective or microcirculation enhancing, add to the synergistic effect of the whole. This is referred to as multicomponent-multitargeted therapy. These considerations enable us to understand the pathological processes of diseases by analysing the commonalities in the pharmacological properties of traditional medicinal plant drugs used in multiple cultures to treat the disease. This approach constitutes a new possible use of pharmacognosy, a discipline that has for the past century been dominated by two approaches, the “bench to bedside” and the “bedside to bench” approach.

## The Two Established Hypothesis of Medicinal Research

Both the “bench to bedside” and the “bedside to bench” approach are based on the application of the ideas of modern biomedicine to the medicinal plants and practices of traditional medicine. In the “bench to bedside” approach, pure chemical compounds are isolated from medicinal plants and tested by high throughput screening for their activity in various *in vitro* model systems as potential drugs.

**FIGURE 1:**
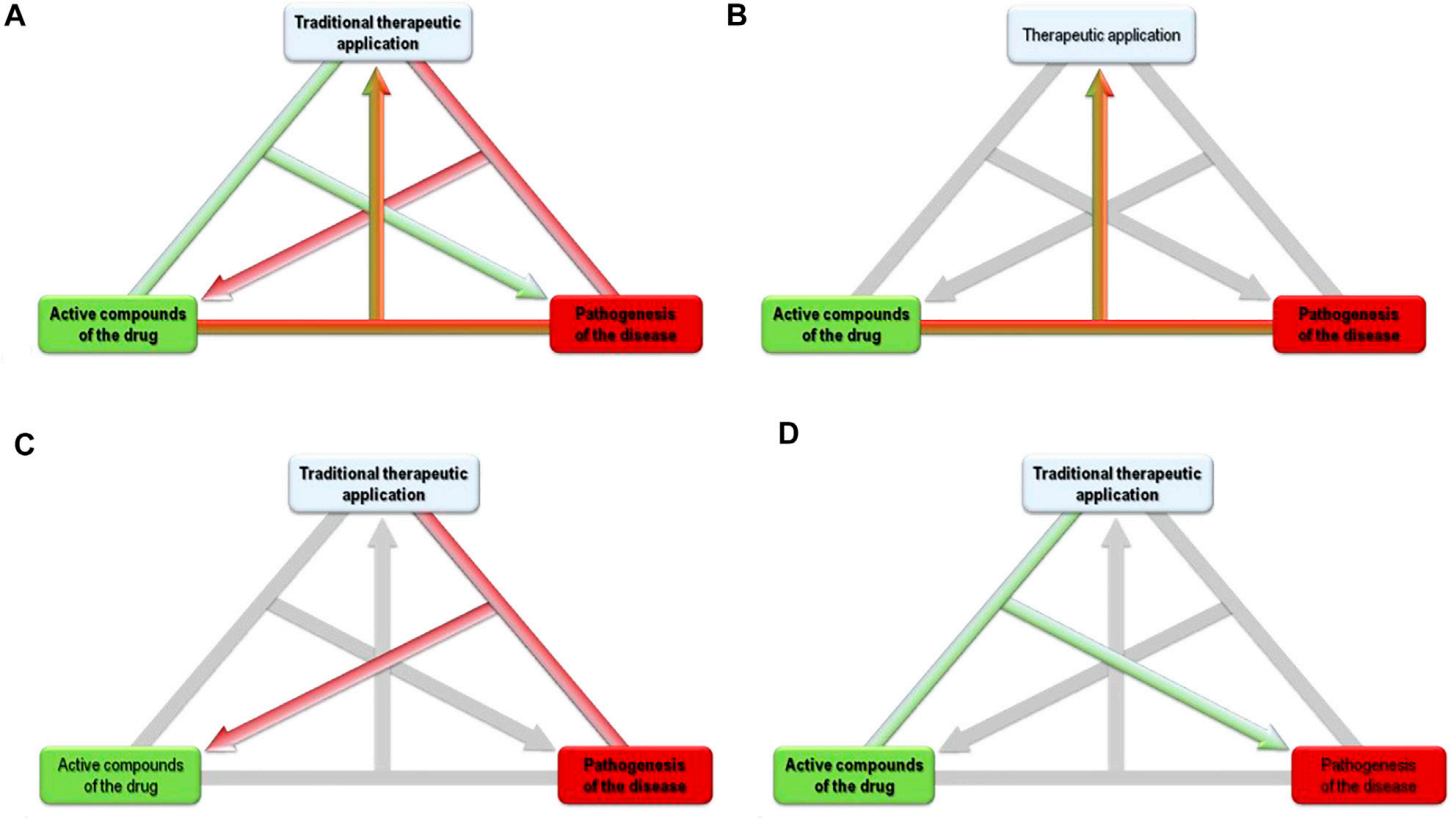
**(A)** From “active compounds of the drug,” to its “traditional therapeutic application,” or “pathogenesis of the disease.” **(B)** Bench to Bedside: Pathogenesis + Active compounds known → Therapeutic applications unknown. **(C)** Bedside to Bench: Pathogenesis + Therapeutic application known → Active compounds unknown. **(D)** Tradition to Pathogenesis: Active compounds + Therapeutic application known → Pathogenesis unknown.

The alternative “bedside to bench” approach uses modern biochemistry and pharmacology in order to characterize traditional medicinal plant preparations and their acting mechanisms, and to develop refined extracts. Based on this approach, bioassay guided fractionation can be applied in order to develop advanced herbal medicinal products with improved therapeutic activity (Figure 1).

## Tradition to Pathogenesis: A Third, Novel Hypothesis of Medicinal Research

Here, we propose a third way of medicinal plant research: We propose that the traditional use of botanical drugs may clarify the pathogenesis of modern diseases. The known bioactivities of the plant constituents and their traditional application might thus help to understand the pathological processes of the treated disease. Figure 1 intends to visualize that if two of the three items “traditional therapeutic application,” “active compounds of the drug” and “pathogenesis of the disease” are known, the third can be researched based on the other two. E.g., in “bench to bedside,” a compound and its pharmacology are known, the research aims to find a fitting therapeutic application. In “bedside to bench,” the traditional therapeutic application and the pathophysiology of the treated disease are known, research aims to find the respective active components (of the plant extract). Our new proposal completes the logical triangle by using the known traditional therapeutic application of the plant - and its known compounds with known activities - for research that aims to find the pathophysiology of the treated disease. This approach can thus be called “Tradition to Pathogenesis” in line with the two previously established approaches. The knowledge of medicinal plants thus helps to understand the shared pathogenesis or association of different diseases and the association between traditional and modern biomedical based pathogenesis.

The idea of predicting the source of evolutionary pressure from an observed adaptation is not new. In 1862, Charles Darwin predicted that the 40 cm long nectary of the Madagascan orchid *Angraecum sesquipedale* Thouars indicated that there must be a pollinating insect with an equally long proboscis (Darwin, 1862). This was confirmed in 1903 when the sphinx moth *Xanthopan morganii praedicta* was discovered by W Rothschild and K Jordan. Here, we apply the same line of reasoning to the cultural evolution of medicine for the first time.

## Learning From History

### *Helicobacter pylori* as the Causative Agent of Gastric Ulcers

As one example, in the 1980s, it was found that the bacterium *Helicobacter pylori* can cause peptic ulcers - a discovery honoured with the 2005 Nobel Prize in medicine to JR Warren and BJ Marshall (Marshall et al., 1985). This result could have been anticipated as numerous systems of traditional medicine worldwide treat peptic ulcers with herbal drugs that exert pronounced anti-bacterial properties.

**FIGURE 2:**
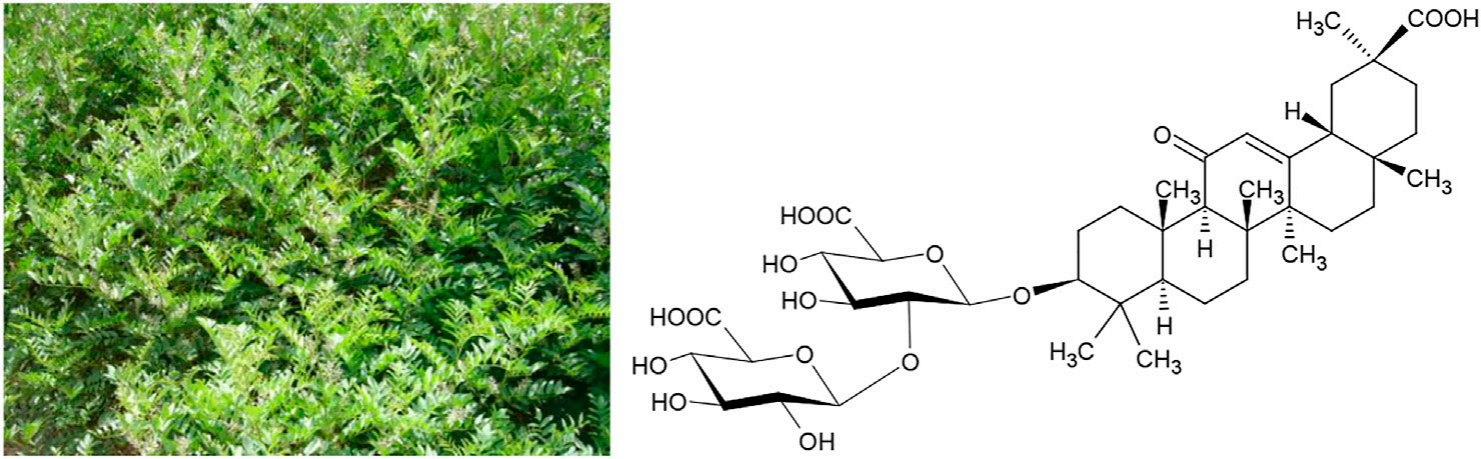
*Glycyrrhiza uralensis* Fisch.exDC. and its active constituent glycyrrhizic acid.

One amongst several such examples are the plant drugs from *Glycyrrhiza spec.* (*G. glabra* L. and *G. uralensis* Fisch.exDC.) that are used as remedies against peptic ulcers from the Atlantic to the Pacific, and the anti-bacterial activity of which is well documented in the literature (Verheijen, 1948). Recent experimental work has verified the effectiveness of *Glycyrrhiza spec.* extracts against *Helicobacter pylori* (Asha et al., 2013). Typical active constituents of *Glycyrrhiza spec.* are triterpene glycosides like saponins such as glycyrrhizic acid (Figure 2).

**FIGURE 3:**
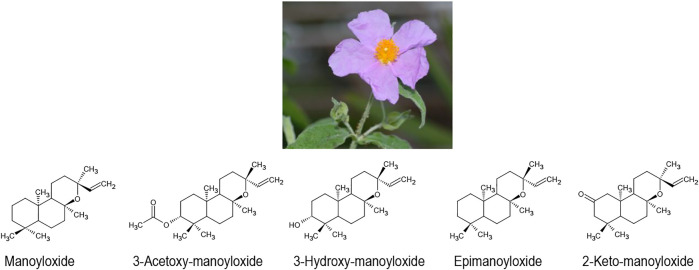
*Cistus creticus* L. and its active manoyloxides.

Another famous plant drug against gastric ulcers is the Mediterranean species *Cistus creticus* L. (including some other species of the same genus). In Cretan traditional medicine, small clumps of labdanum resin, which is collected from the leaves of the plant, are swallowed with Raki as a traditional treatment for gastric ulcer (oral communication, Nyktaris Dimitris, Crete). In Turkish and Italian traditional medicine, tee infusions of the flowers and leaves are used in the same indication and have been successfully tested in animal models (Attaguile et al., 1995; Yesilada et al., 1997). Direct activity of *Cistus spec.* extracts on *Helicobacter pylori* cultures *in vitro* have also been reported (Yesilada et al., 1999). However, in the ancient East Mediterranean, the plant found much wider uses as incense, anti-infective, and for wound treatment (Husemann, 1889; Zohary, 1983). Most recently, a strong activity of the volatile oil phase of the extract against *Borrelia burgdorferi in vitro*, could be demonstrated (Hutschenreuther et al., 2010; Kuchta et al., 2012; Rauwald et al., 2013). This volatile oil is mainly characterised by manoyloxides such as (manoyloxide, 3-acetoxy-manoyloxide, 3-hydroxy-manoyloxide-epimanoyloxide, 2-keto-manoyloxide), (Figure 3) (Kuchta et al., 2012).

In our previously published research (Rauwald et al., 2019), we were able to show that these manoyloxides are specific for labdanum from Cretan *C. creticus*. Besides monoterpenes, simple alkanes were dominant in Spanish labdanum - traditionally prepared by hot water extraction of the aerial parts of *Cistus ladanifer* L., whereas only traces of the major anti-bacterial and anti-viral manoyloxide constituents could be detected (Rauwald et al., 2019). This corresponds with the historical development of the use of labdanum in European traditional herbal medicine: After the Ottoman conquest of Crete in 1645, Western European doctors shifted from Cretan labdanum to Spanish labdanum for the treatment of infectious diseases. Shortly thereafter, labdanum largely fell out of pharmaceutical use in most areas outside of the natural range of *C. creticus* (Rauwald et al., 2019). Labdanum of *Cistus ladanifer* L. continues to be used for perfume.

Apart from marker compounds of medicinal plant drugs, we also have “markers” for judging treatment response. The aim of medical treatments has changed from “absence of symptoms” in traditional medicine to “absence of analytical marker compounds or organisms in the human body” today. Consequently, a direct comparison of antibacterial *in vitro* effects of plant extracts (based on historical documents) with that of mono-molecular antibiotics will often give confusing results. In the case of *H. pylori*, about one third of the world population test positive for its presence in the stomach, although only a small fraction of these will ever develop gastric ulcer. In the vast majority of cases, *H. pylori* remains present but inactive. As traditional healers had no means of detecting the bacteria in the human organism, they could only judge the success of their therapy based on the symptoms of their patients. Consequently, the restoration of this inactive, symptom free state was the adaptive peak in the cultural evolution of traditional medicine.

Based on the above example of *H. pylori*, we have collected data on two other diseases where the apparent mechanism of action of traditional herbal medications across a multitude of cultures should help to clarify the underlying pathogenesis.

### Rheumatoid Arthritis and Spirochaeta Infection

One of the most interesting and most consistent correlations between seemingly unrelated traditional indications of medicinal plants in numerous human cultures is the correlation between syphilis and rheumatoid arthritis.

**FIGURE 4:**
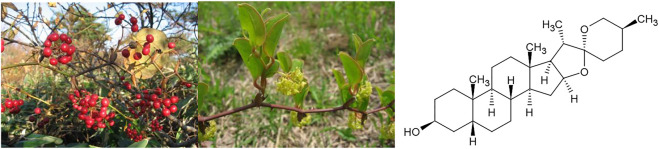
*Smilax china* L. and its active constituent sarsasapogenin.

For example, in Japanese Kampo medicine, the main indication for *Smilax china* L. is syphilis. The same is true for its most common formulation “Hachimitaigeho,” which is further used against numerous infectious and inflammatory diseases of the female reproductive system (Otsuka et al., 2016). In addition, in the Chinese “Bencao Gangmu,” the most extensive and famous compendium of classical Chinese drugs, rheumatoid arthritis is mentioned as a secondary indication (Luo, 2003). Its most significant active components are triterpenes like sarsasapogenin (Figure 4).

We find both indications in Western Herbal Medicine, where the Mesoamerican species of the same genus, mainly *Smilax aristolochiifolia* Mill. (syn. *S. medica* Schltdl.&Cham.) and *Smilax officinalis* Kunth are used. In the Eclectic Medicine Tradition of the United States but also in Europe, these species were used as the preferred herbal remedy for syphilis, especially in the chronic stage of the disease, and also highly recommended for the treatment of rheumatic affections (Felter and Lloyd, 1905; Pereira and Brown, 1855). It is interesting to note that the British pharmacognosist Jonathan Pereira, one of the most famous of his time, already theorised a hidden “venereal origin,” i.e., syphilis infection, as a possible cause of rheumatism (Pereira and Brown, 1855) and Felter and Lloyd (Felter and Lloyd, 1905) also mention “gonorrhoeal rheumatism.”

**FIGURE 5:**
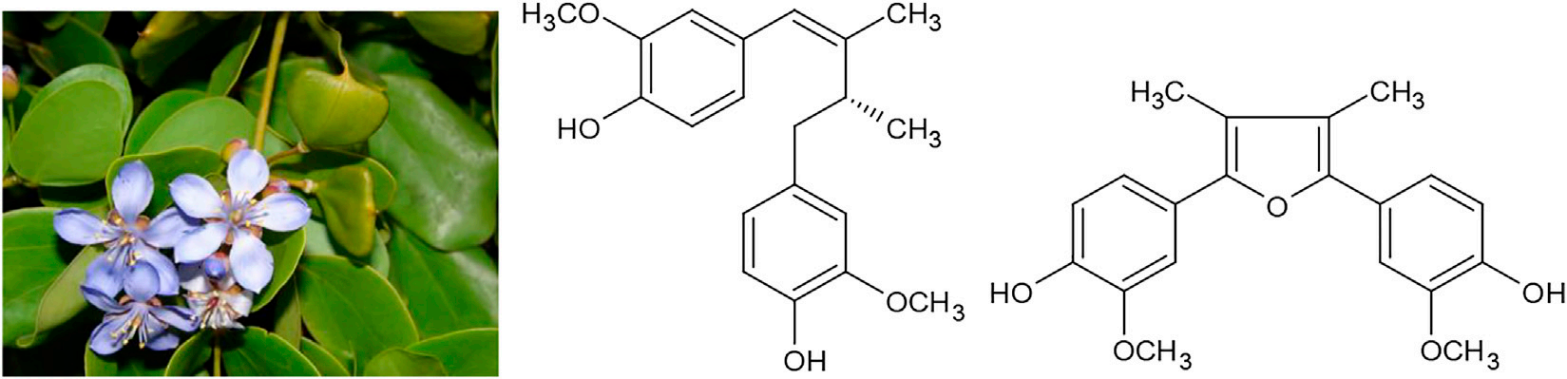
*Guaiacum officinale* L. and its active constituents dehydroguaialignan and furoguajacin.

Another medicinal plant that was intensively used against syphilis is the so called “lignum vitae,” the resin or alcoholic extract of the wood of the Caribbean trees *Guaiacum officinale* L. or *Guaiacum sanctum* L. After the epidemic spread of syphilis through Europe in the 15th century, these drugs were imported in large quantities and praised for their effectiveness in the treatment - or at least suppression - of the disease. One of the first patient narratives in the history of medicine were the treaties “De morbo Gallico” (1519) by the German knight and scholar Ulrich von Hutten, who suffered from syphilis himself and described his own treatment (von Hutten, 1533). Among the therapies von Hutten described, *Guaiacum* resin seems to have been the most effective. Soon, the same drug also found use against rheumatoid arthritis and even as late as 1907, the “British pharmaceutical codex” of the Pharmaceutical Society of Great Britain describes an alkaline solution of *Guaiacum* resin which “use is empirical in chronic rheumatism, rheumatoid arthritis, and syphilis” (Pharmaceutical Society of Great Britain and Council: University College, 1907). Major components of *Guaiacum* resin are lignans like dehydroguaialignan and furoguajacin (Figure 5).

**FIGURE 6:**
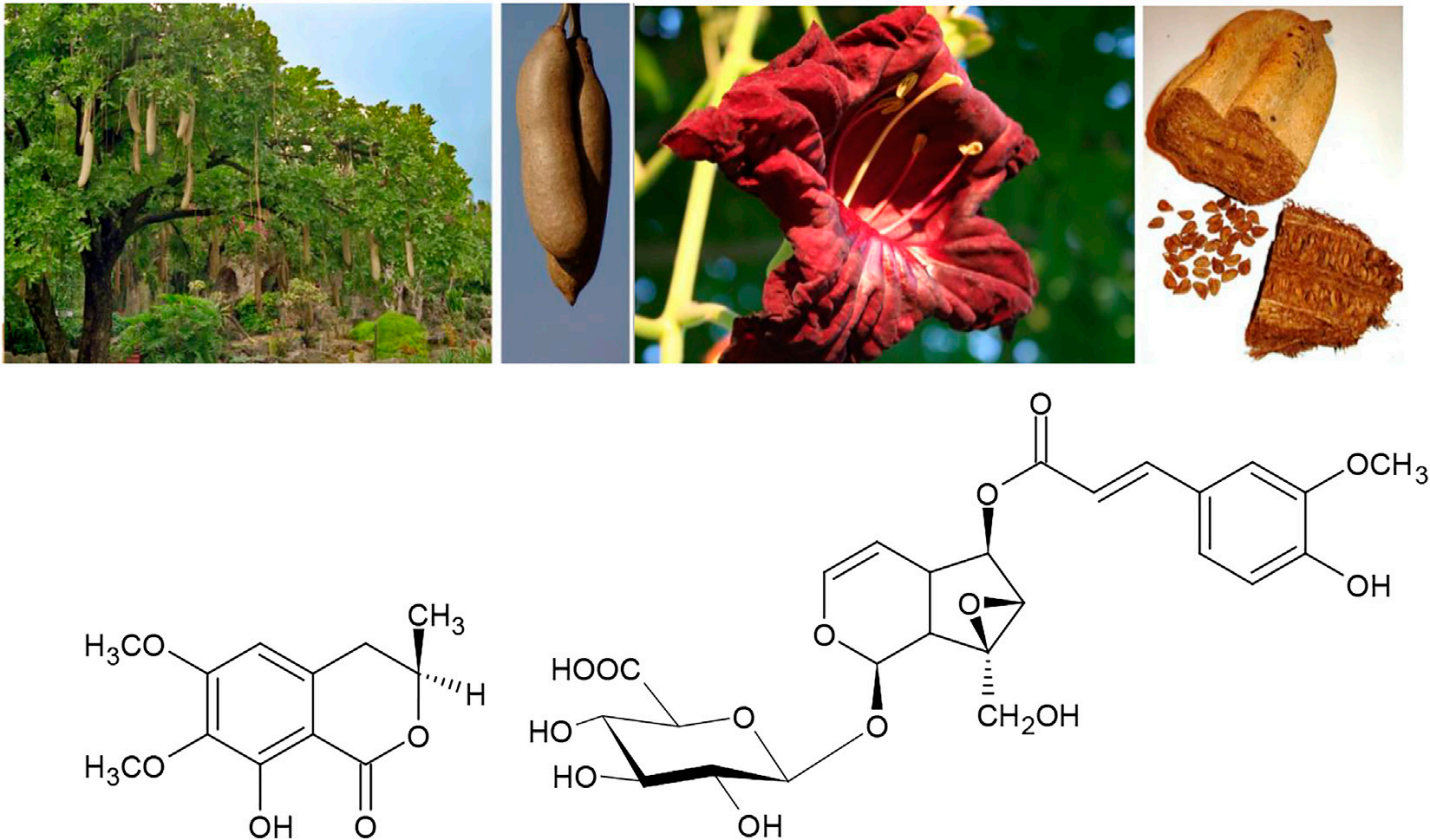
*Kigelia africana* (Lam.)Benth. and its active constituents kigelin and minecoside.

Turning to African traditions, a considerable amount of medicinal knowledge has been documented during the last two centuries. Also in this tradition, an example of the dual use of the same medicinal plant against both, rheumatoid arthritis and syphilis features very prominent: “Both the unripe fruits and the bark of the sausage tree *Kigelia africana* (Lam.)Benth. are taken as a traditional remedy for syphilis and rheumatism” (Neuwinger, 1996; Orwa et al., 2009). Potentially active constituents are kigelin and minecoside (Figure 6). *K. africana* has also been shown to interfere with the response of bacteria to quorum sensing autoinducer compounds that inform the microbes about the density of their own population in several types of bacteria. This mechanism therefore facilitates the manipulation of bacterial growth speed making it a promising candidate for developing the ancestral knowledge of Traditional African medicine to a future in the context of integrative medicine (Kahumba et al., 2015).

### Bacterial Translocation Through the “Leaky Gut” and the Pathogenesis of “Autoimmune Diseases”

The above described cultural evolution of therapeutic procedures does not only apply to the traditional use of medicinal plants but also to empirical experience in the use of modern medicine. In this context, interesting observations concerning the therapy of chronic diseases of the bone and joints in correlation with potential gastrointestinal infections have been reported.

**FIGURE 7:**
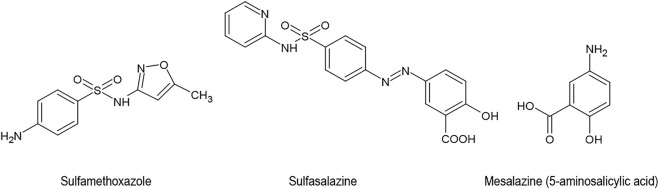
Sulfamethoxazole Sulfasalazine Mesalazine (5-aminosalicylic acid).

Sulphonamides are a class of antibiotic agents that were developed in Germany during the 1930s. They suppress the enzyme dihydropteroate synthase and inhibit the incorporation of para-aminobenzoic acid into folic acid. The affinity of sulphonamides for the bacterial enzyme is about 10,000 times greater than its affinity for the corresponding mammalian enzyme. However, the sulphonamide sulfamethoxazole, which was introduced to the US in 1961 as a remedy for bacterial infections such as urinary tract infections and bronchitis, soon developed a secondary, purely empirical career in the therapy of alleged “autoimmune diseases” and especially osteoarthritis (Rozin, 2007). These observations are not limited to sulfamethoxazole. The related sulfasalazine is also capable of suppressing clinical symptoms and biochemical signs of rheumatoid arthritis (Neumann et al., 1983; Pullar et al., 1983). Sulfasalazine can further be used for joint-pain associated with inflammatory bowel disease. Its use has however declined because of side effects (Voulgari, 2011). Instead, 5-aminosalicylic acid (mesalazine) is used, which is devoid of the antibacterial sulphonamide group in the molecule (Figure 7).

These empirical developments of the clinical use of chemosynthetic agents - a short term “cultural evolution” if you like - have already repeatedly led to publications of theories proclaiming the bacterial origin of rheumatism (Rozin, 2007; Neumann, 1988). These theories could however not enter the clinical mainstream as bacterial products could not yet be detected in the liquid after joint puncture. In this context of inflammation and intractable bacterial infection, spontaneous bacterial peritonitis in liver cirrhosis is one of the rare cases where standard therapy consists of broad-spectrum antibiotics, even though no bacterium is found in the ascites. For diagnosis, abdominal pain and elevated leucocyte or neutrophil count within the ascites are sufficient. As for Crohn’s disease, NOD2 variants are genetic risk factors for bacterial translocation (Appenrodt et al., 2010).

Bacterial translocation is also of highest interest in relation to the so called “leaky gut” syndrome, which is currently at the centre of the scientific discussion concerning the pathogenesis of diverse “autoimmune diseases.” In pathologic conditions, the permeability of the gut epithelial lining can be compromised allowing the passage of toxins, antigens, and bacteria in the lumen to enter the blood stream creating a “leaky gut.” Commensal bacteria from the gut lumen are able to escape from a “leaky gut” together with their products, inducing inflammation and even systemic tissue damages if translocated into peripheral circulation (Brenchley and Douek, 2012). The increased membrane permeability of the intestinal mucosal barrier appears to further correlate with a host of clinical disorders including: inflammatory and functional bowel disease, food allergies, allergic disorders, rheumatoid arthritis, celiac disease, and chronic dermatological conditions (Porras et al., 2006; Zhou et al., 2009).

All the above hints strongly to an antibacterial therapy regime as a promising treatment approach. Further treatment considerations should include modulation of the intestinal flora or mucosal protection, both of which are available in herbal therapies such as *Glycyrrhiza glabra* (Asha et al., 2013), *Cistus* spec. (Attaguile et al., 1995; Yesilada et al., 1997), or the Japanese Kampo prescription Juzentaihoto (Otsuka et al., 2016).

### The Cultural Evolution of “Off Label Uses” for Active Constituents

**FIGURE 8:**
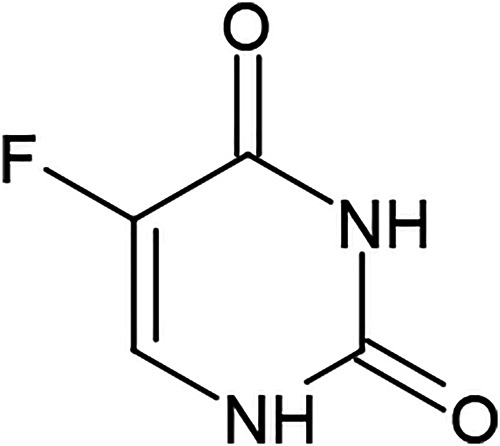
Fluoruracil (5-FU).

The observation already described by Darwin (Darwin, 1871) that evolutionary processes - replication, mutation, and selection of information - occur throughout all aspects of human culture poses the question whether the historical repurposing of a medicines - as proposed above i.e., for sulphonamides - can be compared with the development of traditional medicine through cultural evolution. This includes many cultural practices such as therapeutic applications and can - also in the case of synthetic drugs - lead to the development of “off label uses” *via* the described evolutionary processes. Their empirical use can evolve in the same way as for herbal remedies, a form of “short-term cultural evolution” that shall serve here as an introductory example: 5-FU is a chemotherapeutic agent that acts as a wrong base analogue based on its structural similarity with the pyrimidine base cytosine and thymidine (DNA) and uracil (RNA). It has been used as therapy for gastrointestinal cancers since the 60s (Figure 8). Whereas most cases show tumour regression upon 5-FU therapy, some cases react to this agent with - as of yet unexplained - complete tumour remission.

In order to understand these varying response rates, we might look at alternative uses for 5-FU, namely as a mutagenic agent for RNA viruses. The antiviral activity of 5-FU against the lymphocytic choriomeningitis virus was demonstrated in an animal model and has been interpreted to predict a potential efficacy for other arenaviruses, such as Lassa fever (Ruiz-Jarabo et al., 2003). As 5-FU boosts the rate of mutations *via* incorporation during viral RNA synthesis it is a prime candidate for research into approaches for anti-viral therapy based on the quasi-species model of RNA virus evolution. This effect has been experimentally demonstrated, e.g., for influenza viruses (Pauly and Lauring, 2015). As 5-FU is an anti-mitotic agent which also inhibits thymidylate synthase, it further prevents DNA synthesis. In this context it has been shown that for noncancerous manifestations of human papilloma virus (HPV), a group of DNA viruses of which certain strains cause common warts (verruca vulgaris), local application is effective (Kollipara et al., 2015).

The quasi-species model (Eigen, 1971; Eigen, 1996) proposes that it should be possible to cure viral infections by boosting the mutation rate of the viral genome above the selection rate of the surrounding evolutionary pressure. Above this error threshold (Biebricher and Eigen, 2005), nonsense mutants are generated faster than they can be selected against, resulting in a meltdown of the genetic information that is referred to as “error catastrophe” (Eigen, 2002).

The bench to bedside research on potential therapeutic agents that target the viral “error catastrophe” has not yet resulted in approved drugs for clinical use. However, newer research into human resistance mechanisms against HIV-AIDS indicate that the human immune system itself applies precisely this antiviral strategy in form of the APOBEC3G protein that initiates an increased mutation rate in the viral genome (Malim, 2009). This mechanism appears to be evolutionarily conserved and not just active against HIV but also hepatitis B virus, simple retroviruses, and even endogenous retroelements (Holmes et al., 2007).

Can we therefore assume, that those gastrointestinal tumours which quickly and completely recede under 5-FU might be caused by viral infection at the initial stages?

Could the viral “error catastrophe” or the APOBEC3G system be a potential target of traditional antiviral plant drugs with as of yet unknown mechanisms of action?

Similar reasoning also applies to numerous traditional medicinal plants and their use.

One of the most impressive examples is certainly *Artemisia annua* L. and its constituent artemisinine. Based on the traditional application of the fresh plant juice in the treatment of malaria as documented in the Zhou Hou Jiu Zu Fang (A Handbook of Formulas for Emergencies), written in 340 AD by the Chinese physician and Daoist philosopher Ge Hong, Tu You-you et al. (You-you, 1982a; You-you et al., 1982b) isolated the active sesquiterpene lactone artemisinine, that has since been marketed worldwide as a treatment for malaria (*Plasmodium falciparum*). This research was later honoured with the 2015 Nobel Prize in Physiology or Medicine. After their introduction however, artemisinine and its derivatives like artesunate have proven effective in a number of ailments seemingly unrelated to malaria. These “off label uses” include most prominently the use of *Artemisia annua* and artemisinin for cancer therapy (Efferth, 2017; Efferth, 2006), their activity against viral infections (Efferth et al., 2008) even including hepatitis C virus (HCV) infections (Efferth et al., 2008; Dai et al., 2016), and their ability to attenuate arthritis (Lin et al., 2016). The question, if and how these seemingly unrelated activities are connected and if e.g., viral or protozoal infections might play a role in the development of arthritis might be an interesting starting point for future research.

Galantamine, an alkaloid isolated from *Galanthus woronowii* Losinsk., has shown a similar broad therapeutic versatility. It was originally developed based on the local use in the treatment of poliomyelitis documented in an observational study in the Caucasus Mountains (Heinrich, 2010). In 1951, Mashkovsky and Kruglikova-Lvov (Mashkovsky and Kruglikova-Lvov, 1951) published the first work that establishes the acetyl-choline esterase inhibiting properties of isolated galantamine. Its indication soon broadened to also include myasthenia gravis and muscular dystrophy, residual poliomyelitis paralysis symptoms, trigeminal neurologica, and other forms of neuritis. The scientific rationale for using cholinesterase inhibitors like galantamine in the management of Alzheimer’s disease is based on the cholinergic hypothesis. Impairment of the central cholinergic system is typically observed in Alzheimer’s patients and is accompanied by a loss of cholinergic neurons in the forebrain and a marked decrease in the activity of choline acetyltransferase. Overall, galantamine represents an example for the successful ethnobotanydriven development of a natural product into a clinically important drug (Heinrich, 2010).

### Bone Turnover Related to Improved Testicular Functions

**FIGURE 9:**
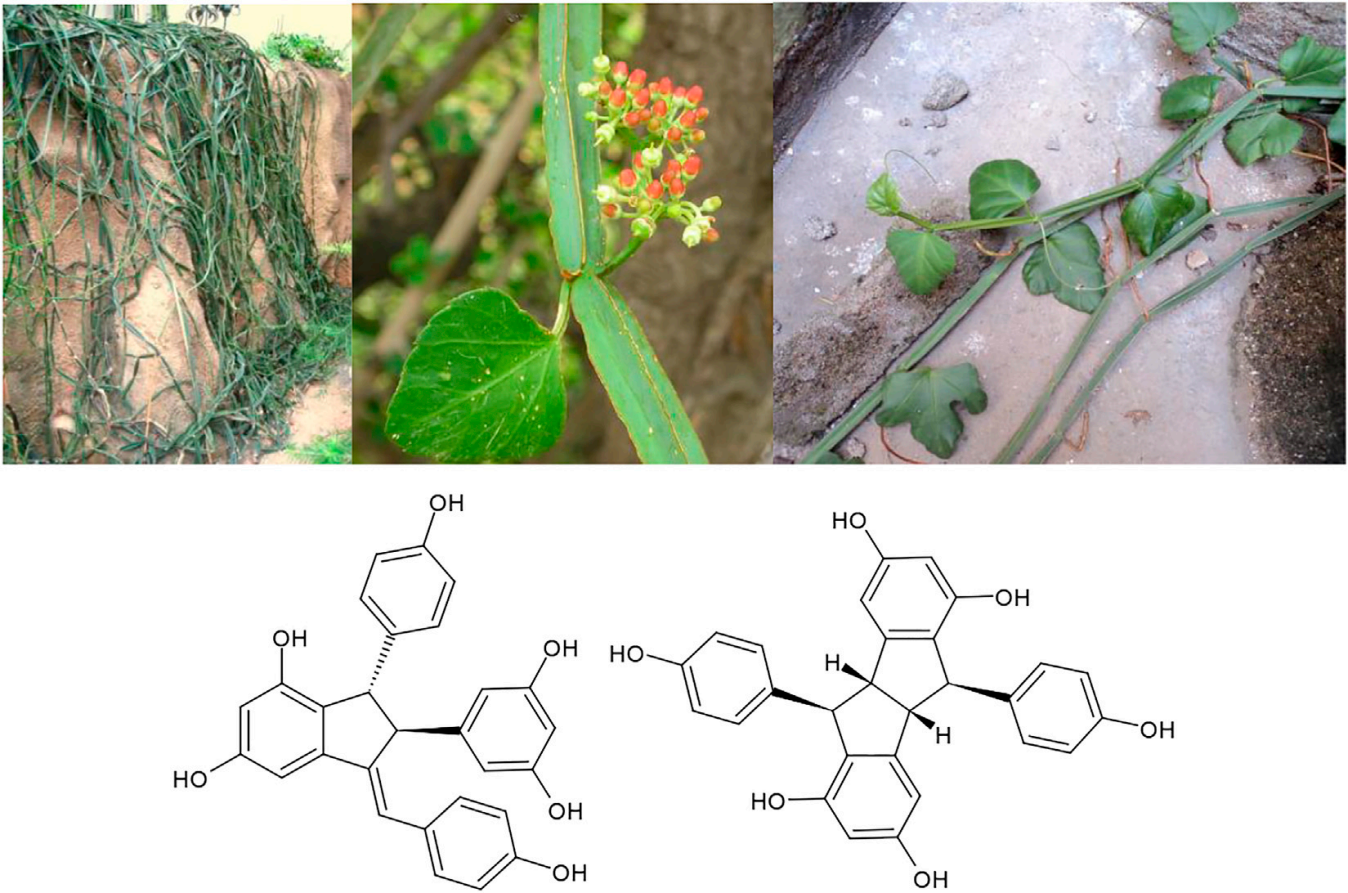
*Cissus quadrangularis* L. and its active constituents quadrangularin A and pallidol.

It is very striking that through numerous medicinal traditions around the world, identical plants are used both for osteological ailments like osteoporosis and for accelerating the healing of broken bones, as well as for the treatment of male sexual dysfunctions like infertility and decreasing sperm production. Whereas numerous “aphrodisiac” effects are reported from local medicine worldwide, this specific effect is most strongly correlated with the parallel use of the same plant in osteology. E.g., in Sub-Saharan Africa and in India, *Cissus quadrangularis* L. extracts are used in both of these indications (Neuwinger, 1996). Typical constituents of these extracts are stilbenoids like quadrangularin A and pallidol (Figure 9). Extracts of *C. quadrangularis* have been experimentally demonstrated to accelerate the healing of fractured jaw bones in an animal model (Brahmkshatriya et al., 2015), to alleviate bone deterioration in osteotomized rats *via* p38 MAPK signalling (Kanwar et al., 2015), to up-regulate the matrix mineralization of human osteoblast like SaOS-2 cells (Muthusami et al., 2011), and to enhance biomineralization through up-regulation of MAPK-dependent alkaline phosphatase activity in osteoblasts (Parisuthiman et al., 2009). The validity of the traditional use of the same plant for increasing sperm production has also been experimentally validated (Neuwinger, 1996). Most recently, extracts of *C. quadrangularis* were demonstrated to prevent quinalphos induced male reproductive toxicity in an animal model (Kokilavani et al., 2014). For the similarly used closely related species *Cissus populnea* Guill.&Perr. a proliferation effect on the sperm producing TM4 Sertoli cell line was observed (Osibote et al., 2011).

**FIGURE 10:**
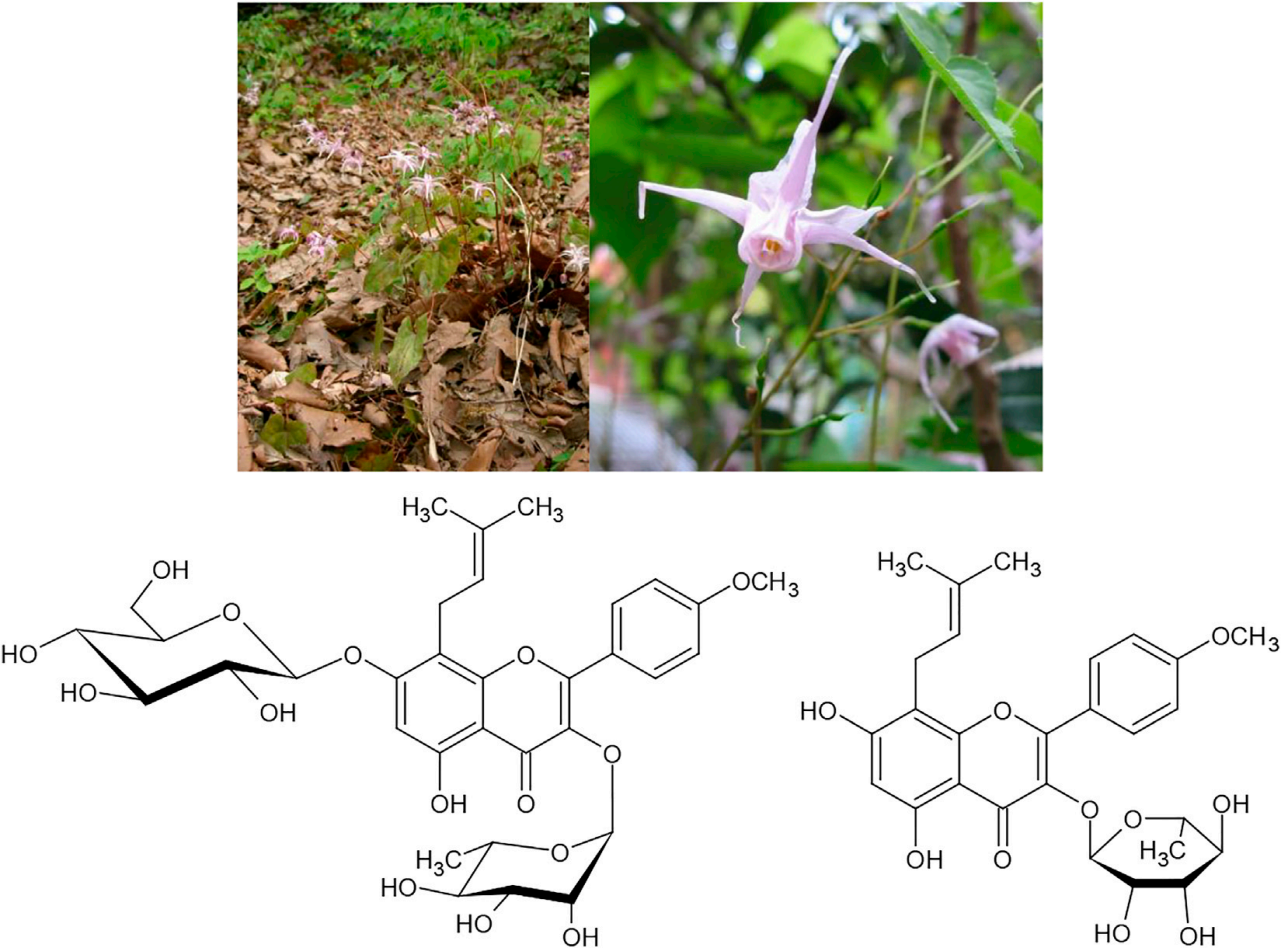
*Epimedium grandiflorum* C.Morrenv and its active constituents icariin and icariside II.

In East Asia, we find several medicinal plant drugs with the same pattern of dual traditional use. Especially for species of the genus *Epimedium* (e.g., *E. grandiflorum* C. Morren) numerous uses in both indications are known. A review of its uses in osteoporosis therapy has been published by Zhai et al. (Zhai et al., 2013). It was shown to induce bone neoformation, to reduce osteocyte and osteoclast densities (Burim et al., 2016) and to induce osteogenesis from bone marrow mesenchymal stem cells (Kim et al., 2017). These effects have mainly been attributed to its prenylated flavonol glycoside icariside II, which can enhance the osteogenic differentiation of bone marrow stromal cells (Luo et al., 2015), and icariin, which modulates the process of bone formation *via* the BMP-2/Smad4 signal transduction pathway (Figure 10) (Liang et al., 2012). Furthermore, in a mouse model, significant increases of testicular weights, sperm counts and sperm motility were observed under treatment with the total flavonoid fraction of the drug (Yuan et al., 2014). In an *in vitro* model, isolated icariin was shown to promote the proliferation of Sertoli cells by activating the ERK1/2 signal pathway. In a parallel study the prenylflavonoid (i.e., icariin derivatives) fraction of an extract of leaves of the closely related species *E. koreaum* was shown to exert powerful protective effects on ovariectomy induced osteoporosis in rats by stimulating bone formation and inhibiting bone turnover and bone resorption, suggesting that the extract fraction could be an alternative to hormone replacement therapy for the prevention of postmenopausal osteoporosis (Zhao et al., 2016).

The above discussed case of the application of *Epimedium* spec. for both osteoporosis and sexual dysfunction reflects the symptom pattern in traditional East Asian medicine systems referred to as “decreased kidney function.”

**FIGURE 11:**
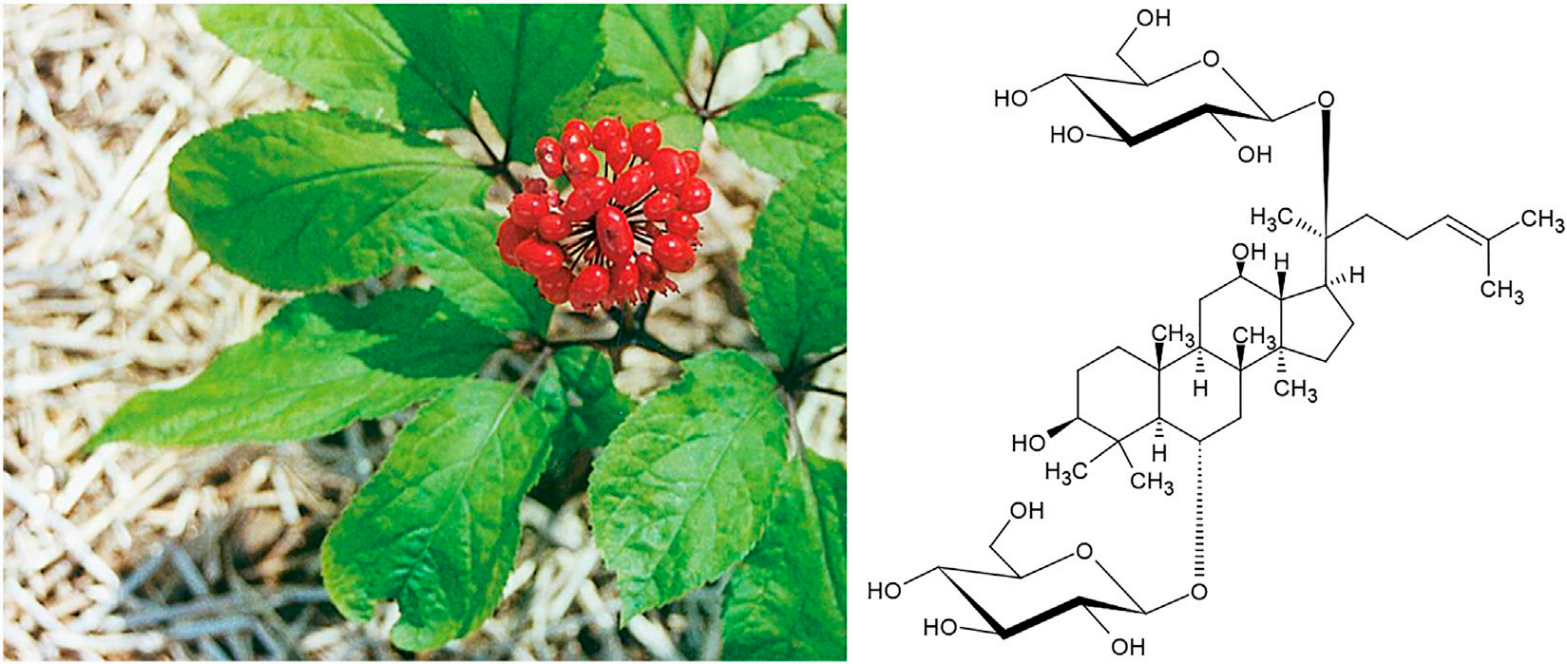
P*anax ginseng* C.A.Mey and its representative active constituent ginsenoside Rg1.

Yet another East Asian plant drug for which this dichotomy of indications can easily be observed is *Panax ginseng* C.A.Mey (Figure 11). In both, *in vitro* and animal models Korean Red Ginseng was shown to prevent radiation-induced bone loss (Kim et al., 2015) and to counteract glucocorticoid-induced osteoporosis (Lee et al., 2013). In a trial with elderly rats, sperm number, germ cell count, Sertoli cell count and Sertoli cell index were significantly restored (Kopalli et al., 2015). In similar experiments, a significant increase of testicle weight was observed (Kim et al., 2010) and testicular damage through 2,3,7, 8-tetrachlorodibenzo-p-dioxin was minimised (Kim et al., 1999).

One possible explanation for the described twofold activity of numerous plant drugs on both the bone and the testicular system may lie in the close ontological relation between the testicular cells and the bone marrow cells in vertebrate ontogenesis (Nayernia et al., 2006). This is especially true for bone marrow cells, in the case of which a direct effect of *P. ginseng* extracts has been experimentally observed (Kim et al., 2014).

The testicular functions are mainly regulated *via* androgenic hormones like testosterone (Kopalli et al., 2015), whereas the formation of bone tissue depends on the p38 MAPK signalling (Kanwar et al., 2015). Recent research points to a regulatory connection between the early steps of these two pathways (Jin et al., 2010; Banerjee et al., 2016; Chen et al., 2016). It therefore seems likely that the traditionally used plant extracts interact with pharmacological targets early in the regulatory pathways. However, experimental data in this regard are sparse and much further research is needed.

The presented insights into this field may not only give us a deeper understanding of male reproductive problems but also lead to “new” approaches in the treatment of common osteological diseases like osteoporosis and further to adjuvant treatments for bone fractures. Last but not least, the possibility of prevention of bone loss may be of interest for deep space travel as a pharmaceutical approach to prevent the ensuing bone loss from microgravity.

## Conclusion

Based on the above presented series of documented cases of dual use of the same medicinal plant for seemingly unrelated diseases from various systems of traditional medicine worldwide, as well as by theoretical considerations grounded on the principles of biological and cultural evolution, we propose “Tradition to Pathogenesis” as a completely new approach in medicinal plant research: Using the known pharmacological properties of medicinal plants and the documented empirical knowledge of their use, it is possible to gain a new understanding of the pathogenesis of the treated diseases. The study of the “disease symptom patterns” (Chin. Zheng) (Zhao et al., 2015) that are traditionally associated with certain herbal drugs and drug mixtures, may be an important guiding light for future discoveries.

In such traditional medicinal systems like in Traditional Chinese Medicine (TCM), Ayurveda, and Kampo medicine the empirical use of medicinal plants are traditionally not based on the modern knowledge of physiology. However, they contain internally consistent theories of pathogenesis. Anthropologically, these theories of pathogenesis in traditional medicinal systems seem to be based on the accumulation of empirical traditional knowledge over the centuries that was later systematised. Empirical traditional knowledge is - just as any other cultural tradition - subject to cultural evolution. In the context of medicine, this means that successful treatments are remembered and replicated whereas unsuccessful treatment attempts are forgotten or discarded if toxic. However, treatment success depends on 1) human physiology and 2) the pathophysiology of the disease. These two factors form the equivalent of Darwinian evolutionary pressure in the cultural evolution of traditional medicine. Thus - although traditional medicine is not based on the modern knowledge of human physiology, biochemistry or genetics - underlaying information is “imprinted” onto the traditional theories of pathogenesis by cultural evolution. Traditional medical knowledge therefore exhibits an *a priori* internal structure that corresponds to human pharmacology and physiology, long before these were scientifically understood.

The idea that human cultures undergo a similar evolutionary process as genetic evolution goes back at least to Darwin himself (Darwin, 1871). The first dynamic model of gene-culture co-evolution based on Darwin’s principles was published in 1976 by Feldman and Cavalli-Sforza (Feldman and Cavalli-Sforza, 1976). For example, the above discussed case of the traditional application of *Epimedium* spec. for both osteoporosis and sexual dysfunction reflects the symptom pattern in traditional East Asian medicine systems referred to as “decreased kidney function”. I.e., these applications of *Epimedium* spec. have a direct footing in human physiology that was “imprinted” onto the traditional medicine system by cultural evolution. This application of the previously established concept of cultural evolution to traditional medicine and pathophysiology is highlighted in our present work for the first time. It is of note that in modern pathophysiology the suprarenal glands are involved in both bone turnover (cortisol, zona fasciculata) and the production of sexual hormones (zona reticularis).

Whilst the isolation and search for single acting compounds from plants did not lead to a further boost of new chemical drugs, as was seen for salicine, codeine, morphine, digitoxin, irinotecan, vincristine, and taxol, the unlifted treasure lies in the clarification of acting mechanisms of traditional herbal extracts. How the theory of cultural evolution can be applied in order to correlate Traditional to Modern pharmacology is an interesting topic for a wide array of future research. Here, we are focusing on the modern understanding of pathological processes based on traditional views on pathogenesis as a first step towards this aim.
